# Hypertension is associated with an increased risk of peritoneal dialysis–associated peritonitis: a retrospective cohort study

**DOI:** 10.3389/fcimb.2026.1807316

**Published:** 2026-07-16

**Authors:** Shiyuan Zhong, Huiying Liu, Qin Wang, Jiefen Hu, Ju Huang, Yanlin Li, Yu Xiao

**Affiliations:** 1 Zhongshan Hospital of Traditional Chinese Medicine, Zhongshan, China; 2 The Tenth Clinical Medical College of Guangzhou University of Traditional Chinese Medicine, Zhongshan, China

**Keywords:** competing risk, hypertension, peritoneal dialysis, peritonitis, risk factors

## Abstract

**Background:**

Peritoneal dialysis–associated peritonitis (PDAP) remains a major complication of peritoneal dialysis (PD) and is a leading cause of hospitalization, technique failure, and mortality. Hypertension is highly prevalent among PD patients; however, its association with PDAP risk has not been well defined.

**Methods:**

We conducted a retrospective cohort study including adult PD patients followed between January 1, 2007, and December 31, 2024. Clinical characteristics, laboratory parameters, dialysis-related features, and PDAP events were systematically collected. Hypertension was defined by a documented diagnosis, long-term use of antihypertensive medications, or repeated blood pressure measurements ≥140/90 mmHg at baseline. The cumulative incidence of PDAP was assessed using cumulative incidence function (CIF) curves and Gray’s test. Independent associations were evaluated using Fine–Gray competing risk models and multivariable logistic regression analyses.

**Results:**

Among 352 PD patients, 194 (55.1%) had hypertension. During a mean follow-up of 46.1 months, hypertensive patients exhibited a significantly higher cumulative incidence of PDAP. After multivariable adjustment, hypertension remained independently associated with a higher risk of PDAP in both Fine–Gray competing risk models (HR = 4.46, 95% CI 2.49–7.99, *P* < 0.001) and logistic regression analyses (OR = 4.38, 95% CI 2.11–9.08, *P* < 0.001). In descriptive microbiological analyses, hypertensive patients showed a higher proportion of gram-negative organisms, whereas gram-positive organisms predominated in non-hypertensive patients.

**Conclusion:**

Baseline hypertension was independently associated with a higher risk of PDAP in patients undergoing PD. These findings highlight the potential importance of blood pressure status in PDAP risk stratification and long-term PD management.

## 1 Introduction

Peritoneal dialysis (PD) is an important modality of renal replacement therapy for patients with end-stage renal disease. Due to its procedural simplicity, preservation of residual renal function, and flexibility in lifestyle, PD has been widely adopted worldwide (Chabouh et al., 2024). However, peritoneal dialysis–associated peritonitis (PDAP) remains a major complication limiting long-term technique survival and is a leading cause of hospitalization, technique failure, and mortality among PD patients (You et al., 2024). Although the overall incidence of PDAP has declined in recent years due to advances in therapeutic strategies, standardization of aseptic procedures, and improvements in PD-related technologies (Xu et al., 2021), its clinical burden remains substantial. Therefore, further reduction of PDAP risk and improvement of long-term outcomes remain critical challenges in PD management.

Previous studies have identified multiple risk factors for PDAP, including diabetes mellitus, hypoalbuminemia, nutritional status, dialysis-related factors, and patient adherence to PD procedures (Zhang et al., 2025). Hypertension is one of the most prevalent comorbidities in patients undergoing PD. In addition to its well-established cardiovascular effects, hypertension has been associated with chronic inflammation, endothelial dysfunction, microvascular injury, and impaired immune responses. Nishigaki et al. suggested that hypertension-related hemodynamic abnormalities may impair peritoneal microcirculation, promote peritoneal structural remodeling, and weaken local immune defense, thereby increasing susceptibility to infection (Nishigaki et al., 2020). Similarly, findings from Carolyn Guan et al. indicated that the conventional pathophysiological mechanisms linking hypertension to cardiovascular disease—particularly its effects on hemodynamics and vascular function—may provide a theoretical basis for a potential association between hypertension and PDAP (Guan et al., 2022). However, despite the high prevalence of hypertension among patients undergoing PD, its potential role in the development of PDAP has not been well established. Most previous studies have focused on traditional risk factors such as diabetes, nutritional status, and dialysis-related factors, whereas the independent association between hypertension and PDAP remains largely unexplored.

Therefore, we conducted a retrospective cohort study to investigate whether hypertension is independently associated with the risk of PDAP in patients undergoing PD. We further explored the impact of hypertension on PD-related outcomes and compared the microbiological characteristics of peritonitis episodes between hypertensive and non-hypertensive patients. These findings may improve our understanding of the relationship between hypertension and PDAP and provide a foundation for future studies investigating the potential impact of blood pressure management on PDAP risk.

## 2 Materials and methods

### 2.1 Study design

This study was a retrospective cohort study conducted at the PD Center of Zhongshan Hospital of Traditional Chinese Medicine. The study period spanned from January 1, 2007, to December 31, 2024. Adult patients who initiated PD at our center during the study period were included. Baseline demographic characteristics, comorbidities, laboratory parameters, dialysis modalities, and peritoneal function data were systematically collected. All peritonitis events and related clinical outcomes during the follow-up period were recorded. Data were obtained from the electronic medical record system and the PD follow-up database.

All patients were retrospectively identified and followed longitudinally from the initiation of PD until death, transfer to hemodialysis, kidney transplantation, occurrence of peritonitis, or December 31, 2024, whichever occurred first. This study was conducted in accordance with the principles of the Declaration of Helsinki and was approved by the Ethics Committee of Zhongshan Hospital of Traditional Chinese Medicine (approval number: 2024ZSZY-LLK-347).

### 2.2 Study population and eligibility criteria

Patients undergoing PD and managed at the PD Center of Zhongshan Hospital of Traditional Chinese Medicine between January 1, 2007, and December 31, 2024, were included. Clinical data were retrieved from the electronic medical record system and the PD database. Patients were required to meet the following inclusion criteria: (1) adult patients receiving PD at our center; (2) complete medical records, including information on comorbidities, drug allergy history, PD prescription, laboratory parameters, and clinical outcomes; (3) PD duration longer than 3 months to exclude patients in the early technique adaptation period; and (4) age between 18 and 80 years.

The exclusion criteria were as follows: (1) refractory peritonitis, to avoid potential bias caused by repeated therapeutic interventions in patients with special clinical conditions; (2) concomitant active infections other than peritonitis, such as pulmonary, urinary tract, or skin and soft tissue infections; (3) a definite history of benign or malignant tumors; (4) acute cardiovascular or cerebrovascular events within the preceding 3 months, including acute heart failure, acute coronary syndrome, or acute cerebral infarction; and (5) pregnancy. All eligible patients were included in the final analysis, resulting in a total cohort of 352 PD patients.

### 2.3 Data collection and definitions

All data were obtained from the PD center’s electronic medical record system and follow-up database. Demographic characteristics, comorbidities, PD-related information, laboratory parameters, and peritonitis-related data were systematically collected during the study period. Demographic variables included age, sex, educational level, and occupation. PD-related variables included PD modality, PD vintage, peritoneal transport status, PD-related complications, severity of peritonitis, and dialysate glucose concentration. Anthropometric parameters included body mass index (BMI).

Laboratory variables covered biochemical profiles related to nutrition, inflammation, and mineral metabolism, including phosphorus, creatinine, blood urea nitrogen, β_2_-microglobulin, prealbumin, uric acid, hemoglobin, platelet count, ferritin, transferrin saturation (TSAT), potassium, calcium, sodium, carbon dioxide combining power, albumin, parathyroid hormone (PTH), hematocrit, alanine aminotransferase (ALT), aspartate aminotransferase (AST), alkaline phosphatase (ALP), triglycerides, total cholesterol, low-density lipoprotein cholesterol, blood glucose, magnesium, cystatin C, and total protein. All laboratory measurements were obtained from the most recent stable values at baseline, prior to the occurrence of any peritonitis event.

Hypertension was defined using baseline clinical information obtained at PD initiation or study entry. Patients were classified as hypertensive if they met any of the following criteria: (1) a documented physician diagnosis of hypertension in the medical record; (2) long-term use of antihypertensive medications for blood pressure control; or (3) at least two outpatient blood pressure measurements ≥140/90 mmHg recorded prior to or at baseline (Unger et al., 2020). Patients were categorized into hypertensive and non-hypertensive groups based on blood pressure status. Information regarding medication use during long-term follow-up, including antihypertensive agents, lipid-lowering drugs, immunosuppressive agents, corticosteroids, phosphate binders, vitamin D preparations, and erythropoiesis-stimulating agents, was not consistently available throughout the study period and therefore was not included in the present analysis. Patients with missing baseline variables required for multivariable analyses were excluded. All data entries were independently reviewed by two investigators to ensure accuracy and completeness.

### 2.4 Outcomes

The primary outcome of this study was the occurrence of PDAP and its clinical management outcomes. Secondary outcomes included cure, death, PD catheter removal, and transfer to hemodialysis. The diagnosis of PDAP was based on the criteria established by the International Society for Peritoneal Dialysis (ISPD) guidelines and required the presence of at least two of the following three criteria: (1) typical clinical manifestations of peritonitis, including abdominal pain and/or cloudy dialysate; (2) dialysate white blood cell count >100 × 10^6^/L with neutrophils accounting for >50%; and (3) positive dialysate bacterial culture (Al Sahlawi et al., 2022; Szeto and Li, 2019).

All suspected peritonitis cases were confirmed by clinicians based on clinical symptoms, diagnostic examinations, and laboratory evidence. Patients were prospectively followed from PD initiation until the first occurrence of PDAP, death, transfer to hemodialysis, kidney transplantation, or the end of the study period (December 31, 2024), whichever occurred first. For each peritonitis episode, the date of onset, clinical manifestations, laboratory findings, microbiological results, and treatment measures were recorded. The outcome of each episode (cure, catheter removal, death, or transfer to hemodialysis) was documented after completion of the full course of treatment. Considering that multiple competing terminal events may occur during long-term follow-up, PDAP was treated as the primary outcome, while death, catheter removal, and transfer to hemodialysis were considered competing risk events in the statistical analysis. For patient-level analyses, PDAP was defined as the occurrence of at least one episode during follow-up.

### 2.5 Statistical analysis

Continuous variables were assessed for normality using the Shapiro–Wilk test. Variables with a normal distribution were expressed as mean ± standard deviation (SD), whereas non-normally distributed variables were presented as median (interquartile range, IQR). Comparisons between groups were performed using independent-samples t-tests or Mann–Whitney U tests as appropriate. Categorical variables were presented as frequencies and percentages and compared using *χ*² tests or Fisher’s exact tests. The cumulative incidence of PDAP was estimated using cumulative incidence function (CIF) curves, and differences between groups were assessed using Gray’s test. To further evaluate the association between hypertension and the risk of PDAP, univariate logistic regression analyses were first performed to identify potential covariates, which were then entered into multivariable logistic regression models. Variables with a *P* < 0.05 in univariate analysis or those considered clinically relevant based on previous PDAP literature (including age, sex, diabetes mellitus, dialysis vintage, nutritional indicators, and mineral metabolism parameters) were entered into the multivariable model. Results were reported as odds ratios (ORs) with 95% confidence intervals (CIs). Given the presence of competing events such as death, transfer to hemodialysis, and kidney transplantation during follow-up, Fine–Gray competing risk models were applied to estimate the independent association between hypertension and PDAP risk. Subdistribution hazard ratios (HRs) with 95% CIs were reported after multivariable adjustment. Multivariable logistic regression was used to identify factors associated with the occurrence of at least one PDAP episode during follow-up, while time-to-event analyses were further evaluated using Fine–Gray competing risk models. To evaluate potential multicollinearity among covariates included in the multivariable models, variance inflation factors (VIFs) were calculated. A VIF value greater than 5 was considered indicative of substantial multicollinearity. All variables included in the final models showed VIF values below 2, suggesting no significant collinearity among covariates. Differences in pathogen distribution between groups were analyzed using descriptive statistics and *χ*²/Fisher’s exact tests. All statistical analyses were performed using SPSS version 27.0 and R version 4.4.1. All tests were two-sided, and a P value <0.05 was considered statistically significant.

## 3 Results

### 3.1 Participant characteristics

By the end of the study period (December 31, 2024), a total of 352 patients undergoing PD were included in the analysis. Among them, 194 patients (55.1%) had hypertension, while 158 patients (44.9%) did not. The overall mean age was 50.00 ± 11.85 years, with no significant difference between the two groups (*P=*0.850). Males accounted for 55.4% of the cohort, and the proportion of males was significantly higher in the hypertensive group (*P=*0.040). A total of 63 patients (17.9%) had concomitant diabetes mellitus, which was significantly more prevalent among hypertensive patients (*P* < 0.001).

Regarding nutritional and anthropometric parameters, patients with hypertension had a significantly higher body mass index (BMI) compared with those without hypertension (*P=*0.017). Dialysis vintage was significantly shorter in the hypertensive group (*P* < 0.001) (**Table 1**).

**Table 1:** Baseline demographic, clinical, and laboratory characteristics of peritoneal dialysis patients according to hypertension status.

Characteristics	Total (n = 352)	Non-hypertension (n = 158)	Hypertension (n = 194)	P value
Age, years	50.00 ± 11.85	50.13 ± 11.74	49.89 ± 11.97	0.850
Male, n (%)	195 (55.4)	78 (49.4)	117 (60.3)	0.040
BMI (kg/m²)	22.56 ± 3.59	22.05 ± 3.20	22.97 ± 3.83	0.017
Diabetes mellitus, n (%)	63 (17.9)	1 (0.6)	62 (32.0)	<0.001
Education level, n (%)				0.660
Junior high school or below	269 (76.4)	119 (75.3)	150 (77.3)	
High school or above	83 (23.6)	39 (24.7)	44 (22.7)	
Dialysis vintage, months	46.42 ± 38.06	57.14 ± 37.54	37.70 ± 36.29	<0.001
Baseline laboratory parameters				
Phosphorus, mmol/L	1.60 ± 0.49	1.67 ± 0.44	1.55 ± 0.52	0.026
Creatinine, μmol/L	912.76 ± 286.35	937.95 ± 284.46	892.25 ± 286.98	0.137
Blood urea nitrogen, mmol/L	17.27 ± 5.93	17.53 ± 5.75	17.06 ± 6.09	0.457
β2-microglobulin, mg/L	36.61 ± 12.66	36.90 ± 11.54	36.37 ± 13.54	0.704
Uric acid, μmol/L	393.31 ± 94.84	397.48 ± 88.29	389.92 ± 99.96	0.458
Hemoglobin, g/L	120.00 (106.00-131.25)	122.00 (112.25-133.00)	116.00 (101.00-129.75)	0.003
Platelet count, ×10^9^/L	258.00 (205.00-309.00)	259.50 (209.00-303.25)	253.00 (203.00-311.50)	0.434
Ferritin, μg/L	92.20 (40.98-189.65)	101.50 (45.25-184.18)	87.50 (38.10-199.28)	0.424
Transferrin saturation, %	24.02 (16.94-32.65)	25.20 (19.92-35.61)	21.90 (14.78-31.84)	0.005
Potassium, mmol/L	3.86 (3.47-4.29)	3.81 (3.50-4.26)	3.87 (3.46-4.30)	0.745
Calcium, mmol/L	2.29 (2.16-2.45)	2.39 (2.23-2.48)	2.25 (2.10-2.39)	<0.001
Sodium, mmol/L	137.50 (135.00-139.40)	137.60 (135.80-140.00)	137.40 (134.80-139.00)	0.132
Carbon dioxide, mmol/L	27.90 (25.58-29.33)	27.95 (25.80-29.55)	27.85 (25.50-29.28)	0.550
Albumin, g/L	38.40 (35.20-41.13)	39.25 (36.43-41.30)	37.80 (34.10-40.90)	0.011
Parathyroid hormone, pg/mL	206.35 (95.63-355.08)	206.35 (99.35-326.63)	207.50 (93.45-387.65)	0.709
Hematocrit, L/L	0.37 (0.32-0.41)	0.38 (0.34-0.41)	0.36 (0.31-0.40)	0.001
ALT, U/L	16.00 (11.00-22.00)	16.00 (12.25-24.00)	15.00 (11.00-21.00)	0.024
AST, U/L	18.50 (14.00-24.00)	19.00 (15.00-24.00)	18.00 (14.00-23.00)	0.183
ALP, U/L	77.00 (59.00-103.00)	78.00 (60.00-115.00)	73.50 (58.75-100.25)	0.175
Triglycerides, mmol/L	1.43 (1.02-2.14)	1.46 (1.08-2.17)	1.42 (0.93-2.14)	0.443
Total cholesterol, mmol/L	4.51 (3.79-5.36)	4.60 (3.89-5.29)	4.43 (3.69-5.39)	0.248
LDL-C, mmol/L	2.25 (1.67-2.83)	2.30 (1.73-2.83)	2.18 (1.62-2.86)	0.391
Magnesium, mmol/L	0.79 (0.70-0.91)	0.81 (0.72-0.92)	0.79 (0.68-0.90)	0.157
Cystatin C, mg/L	6.43 (5.17-7.18)	6.64 (5.90-7.25)	6.26 (5.52-7.02)	0.010
Total protein, g/L	66.95 (62.40-72.05)	67.05 (64.23-72.20)	66.85 (60.73-71.85)	0.108
KT/V	2.11 ± 0.56	2.18 ± 0.59	2.06 ± 0.54	0.051
PET, n (%)				0.266
High transporter	23 (6.5)	6 (3.8)	17 (8.8)	
High-average transporter	108 (30.7)	47 (29.7)	61 (31.4)	
Low-average transporter	157 (44.6)	71 (44.9)	86 (44.3)	
Low transporter	48 (13.6)	26 (16.5)	22 (11.3)	
Others	16 (4.5)	8 (4.1)	8 (4.1)	
Type of peritoneal dialysis, n (%)				0.706
APD	11 (3.1)	4 (2.5)	7 (3.6)	
CAPD	313 (88.9)	142 (89.9)	171 (88.1)	
CCPD	1 (0.3)	1 (0.6)	0 (0.0)	
PD+HD	19 (5.4)	7 (4.4)	12 (6.2)	
Others	8 (2.3)	4 (2.5)	4 (2.1)	

BMI, body mass index; ALT, alanine aminotransferase; AST, aspartate aminotransferase; ALP, alkaline phosphatase; LDL-C, low-density lipoprotein cholesterol; KT/V, weekly total urea clearance; APD, automated peritoneal dialysis; CAPD, continuous ambulatory peritoneal dialysis; CCPD, continuous cycling peritoneal dialysis; PD+HD, combined peritoneal dialysis and hemodialysis.

Comparisons of baseline laboratory parameters revealed that hypertensive patients had lower levels of hemoglobin, hematocrit, serum albumin, and transferrin saturation. Serum calcium and phosphorus levels were lower in the hypertensive group. In addition, Cystatin C levels were modestly lower in the hypertensive group. No significant differences were observed in other routine biochemical parameters. With respect to peritoneal function and dialysis modality, the distribution of peritoneal transport types was generally similar between groups. Continuous ambulatory peritoneal dialysis (CAPD) was the most commonly used modality, and no significant differences in dialysis modality were observed between hypertensive and non-hypertensive patients (**Table 1**).

### 3.2 Follow-up and outcomes

During a mean follow-up of 46.1 ± 38.3 months, hypertensive patients had a significantly shorter follow-up duration compared with non-hypertensive patients (*P* < 0.001). During follow-up, 89 patients experienced at least one episode of PDAP, accounting for 25.3% of the cohort. A total of 120 PDAP episodes were recorded during the study period. Overall, most episodes of peritonitis were successfully cured (86.6%), while mortality was relatively low (5.1%). The proportions of patients who transferred to hemodialysis (4.0%) or underwent catheter removal due to infection (4.3%) were also low.

When outcomes were compared between groups, no significant differences were observed in cure rate or mortality. However, hypertensive patients were more likely to transfer to hemodialysis (*P* = 0.026), indicating a higher rate of transfer to hemodialysis. Regarding PDAP occurrence, the proportion of patients experiencing at least one episode was significantly higher in the hypertensive group (38.1% vs. 9.5%, P<0.001), indicating a potential association between blood pressure status and susceptibility to infection. Although treatment outcomes (cure, catheter removal, and death) were comparable between groups, the higher incidence of peritonitis and shorter follow-up duration in hypertensive patients reflected differences in long-term prognosis (**Table 2**).

**Table 2:** Follow-up outcomes and occurrence of PDAP according to hypertension status.

	Total (n = 352)	Non-hypertension (n = 158)	Hypertension (n = 194)	P value
Cure	305 (86.6)	139 (88.0)	166 (85.6)	0.533
Death	18 (5.1)	8 (5.1)	10 (5.2)	0.969
Catheter removal	15 (4.3)	9 (5.7)	6 (3.1)	0.291
Switch to HD	14 (4.0)	2 (1.3)	12 (6.2)	0.026
Follow-up time, months	46.1 ± 38.3	57.6 ± 37.6	36.9 ± 36.5	<0.001
All-cause peritonitis	89 (25.3)	15 (9.5)	74 (38.1)	<0.001

HD, hemodialysis.

### 3.3 Factors associated with peritonitis occurrence

To further identify independent risk factors for PDAP, variables with statistical significance or clinical relevance at baseline were entered into logistic regression analyses (**Table 3**). In univariate analysis, hypertension was strongly associated with an increased risk of peritonitis (OR = 5.879, 95% CI 3.208–10.773, *P* < 0.001). Other factors associated with peritonitis included diabetes mellitus (OR = 1.943, *P* = 0.025), lower serum phosphorus (OR = 0.315, *P* < 0.001), lower serum calcium (OR = 0.074, *P* < 0.001), lower serum albumin (OR = 0.868, *P* = 0.010), and lower alanine aminotransferase (ALT) levels (OR = 0.955, *P* = 0.001).

**Table 3:** Logistic regression analysis of factors associated with PDAP.

Variables	Univariate model				Multivariate model			
	*B* value	OR value	95% CI	*P* value	*B* value	OR value	95% CI	*P* value
Male, *n*	0.042	1.043	0.643-1.693	0.864	-0.044	0.957	0.469-1.95	0.903
Age, years	0.016	1.016	0.996-1.037	0.126	0.003	1.003	0.977-1.029	0.82
Hypertension	1.771	5.879	3.208-10.773	<0.001	1.477	4.38	2.113-9.079	<0.001
Diabetes mellitus, *n* (%)	0.664	1.943	1.086-3.476	0.025	-0.596	0.551	0.246-1.234	0.148
BMI (kg/m²)	0.000	1.000	0.935-1.07	0.997	0.007	1.007	0.927-1.095	0.865
KT/V	-0.444	0.641	0.391-1.05	0.078	-0.499	0.607	0.311-1.186	0.144
Dialysis vintage, months	-0.001	0.999	0.993-1.006	0.802	-0.001	0.999	0.991-1.008	0.89
Hemoglobin, g/L	-0.016	0.984	0.972-0.996	0.007	0	1	0.984-1.017	0.989
Transferrin saturation, %	-0.04	0.961	0.939-0.983	<0.001	-0.021	0.979	0.954-1.004	0.098
Phosphorus, mmol/L	-1.156	0.315	0.18-0.55	<0.001	-1.131	0.323	0.159-0.656	0.002
Calcium, mmol/L	-2.603	0.074	0.023-0.238	<0.001	-1.617	0.198	0.047-0.834	0.027
Albumin, g/L	-0.141	0.868	0.825-0.913	<0.001	-0.078	0.925	0.863-0.991	0.028
ALT, U/L	-0.046	0.955	0.929-0.983	0.001	-0.042	0.959	0.928-0.991	0.012

OR, odds ratio; CI, confidence interval; ALT, alanine aminotransferase.

After simultaneous adjustment for relevant covariates in the multivariable model, hypertension remained the strongest independent risk factor for peritonitis (OR = 4.380, 95% CI 2.113–9.079, *P* < 0.001). Serum phosphorus (OR = 0.323, *P* = 0.001) and serum calcium levels (OR = 0.198, *P* = 0.027) continued to show significant protective effects, suggesting a close relationship between nutritional/mineral metabolism status and infection risk. ALT also remained independently associated with peritonitis (OR = 0.959, *P* = 0.012), possibly reflecting the influence of baseline metabolic and systemic conditions on infection susceptibility. Other variables, including diabetes mellitus, BMI, dialysis vintage, hemoglobin, and transferrin saturation, did not retain statistical significance in the multivariable analysis. Overall, these findings indicate that hypertensive patients have a substantially increased risk of peritonitis, independent of other clinical factors, while lower serum phosphorus and calcium levels were independently associated with an increased risk of PDAP (**Table 3**).

### 3.4 Impact of hypertension on the risk of peritonitis

**Figure 1:**
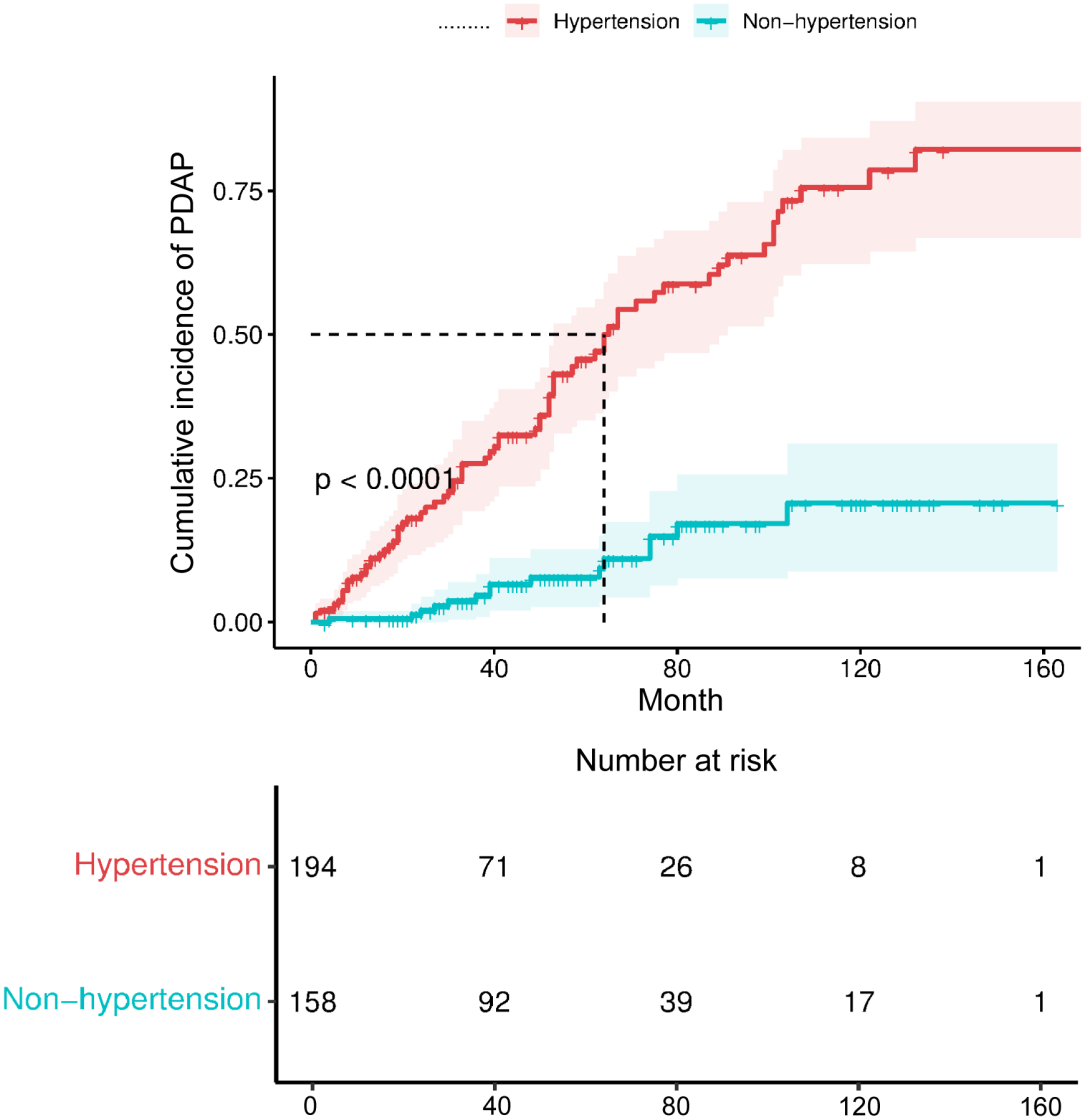
Cumulative incidence function (CIF) curves showing the cumulative incidence of peritoneal dialysis–associated peritonitis in patients with and without hypertension.

To further evaluate the effect of hypertension on PDAP risk, cumulative incidence function (CIF) analysis and Fine–Gray competing risk models were applied. As shown in **Figure 1**, without accounting for competing outcomes, hypertensive patients exhibited a significantly higher cumulative incidence of peritonitis compared with non-hypertensive patients. The CIF diverged early during follow-up and continued to separate over time. Gray’s test demonstrated a significantly higher cumulative incidence of PDAP in the hypertensive group (*P* < 0.001).

**Figure 2:**
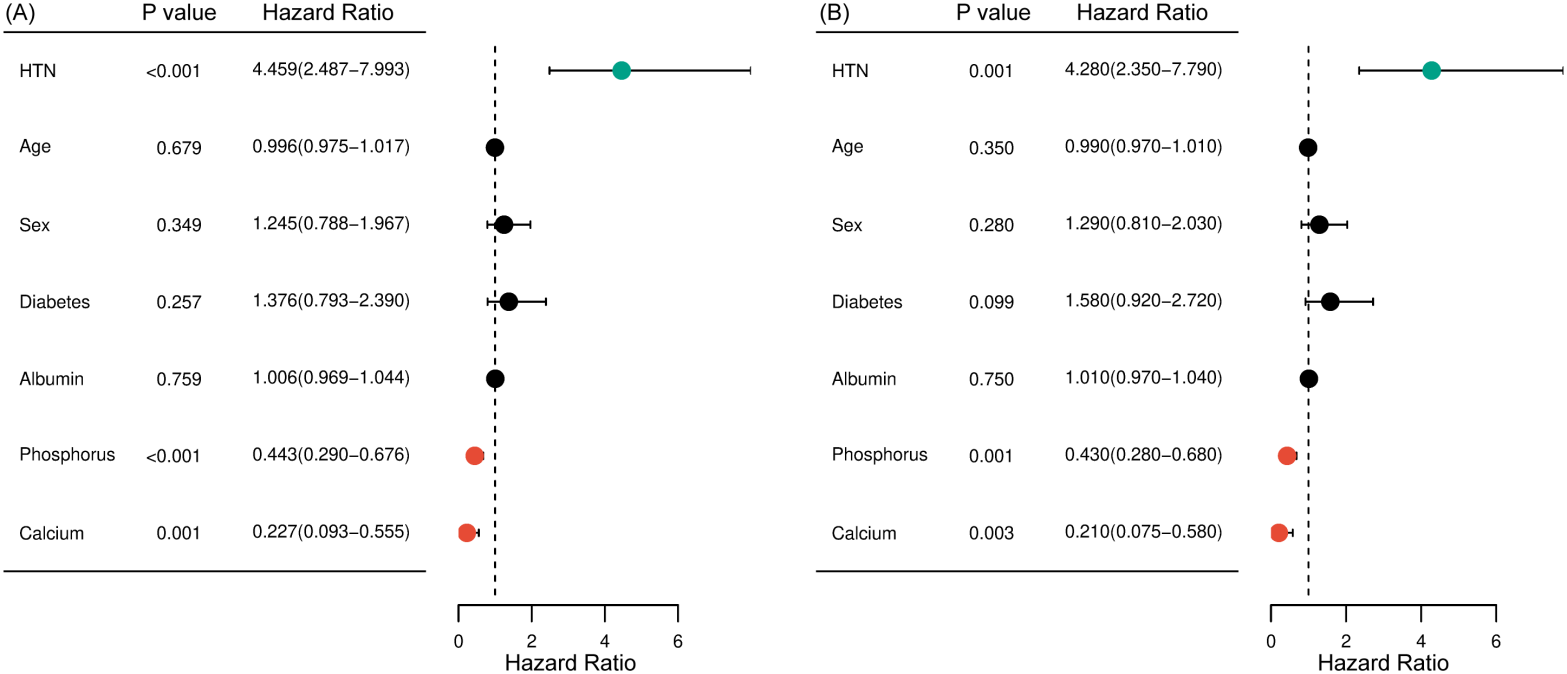
Forest plots from competing risk analyses evaluating the association between hypertension and PDAP. **(A)** Multivariable Fine–Gray competing risk model. **(B)** Sensitivity analysis confirming the robustness of the association.

Considering the presence of competing events such as death and transfer to hemodialysis during long-term follow-up, Fine–Gray competing risk models were subsequently employed. Before multivariable modeling, multicollinearity among candidate covariates was assessed using variance inflation factors (VIFs). All VIF values ranged from 1.08 to 1.27, indicating a low degree of collinearity and supporting the inclusion of these variables in the multivariable analyses. After adjustment for potential confounders, including age, sex, diabetes mellitus, serum albumin, phosphorus, and calcium, hypertension remained significantly associated with an increased risk of peritonitis (HR = 4.46, 95% CI 2.49–7.99, *P<*0.001), consistent with the magnitude observed in the multivariable logistic regression analysis. Serum phosphorus (HR = 0.44, *P* < 0.001) and serum calcium (HR = 0.23, *P* = 0.001) continued to demonstrate significant protective effects, whereas age, sex, diabetes mellitus, and albumin levels were not independently associated with PDAP risk (**Figure 2A**). Sensitivity analyses further confirmed the robustness of these findings (HR = 4.28, 95% CI 2.35–7.79, *P* < 0.001). Regardless of whether models were unadjusted or fully adjusted, hypertensive patients consistently exhibited an approximately a fourfold increased risk of peritonitis, indicating that the association was not materially affected by model specifications (**Figure 2B**). Collectively, these results indicate that hypertension is a strong and independent risk factor for PDAP.

### 3.5 Causative microorganisms of peritonitis and distribution by blood pressure status

The microbiological profiles of all peritonitis episodes are summarized in **Table 4**. A total of 120 peritonitis cases with definite microbiological results were identified. Gram-positive organisms predominated, accounting for 60.8% of cases and representing the most common causative pathogens. *Streptococcus species* were the most frequently isolated organisms, accounting for 39.2% of all cases, followed by coagulase-negative staphylococci (12.5%) and *Staphylococcus aureus* (6.7%). Gram-negative organisms accounted for 19.2% of cases, with *Escherichia coli* (5.8%) and *Pseudomonas aeruginosa* (5.8%) being the most common, followed by *Enterobacter* species (2.5%) and *Klebsiella* species (1.7%). Fungal peritonitis was relatively uncommon (2.5%), while other rare pathogens accounted for 7.5% of cases. Culture-negative peritonitis accounted for 10.0% of episodes. Overall, the microbiological spectrum was consistent with previously reported epidemiological patterns of PDAP.

**Table 4:** Microbiologic causes of peritonitis.

Organisms	Case (*n* = 120)
**Gram-positive organisms**	73 (60.83)
Coagulase-negative *Staphylococci*	15 (12.50)
*Staphylococcus aureus*	8 (6.67)
*Streptococcus* spp.	47 (39.17)
*Enterococcus* spp.	3 (2.50)
**Gram-negative organisms**	23 (19.17)
*Escherichia coli*	7 (5.83)
*Klebsiella* spp.	2 (1.67)
*Enterobacter* spp.	3 (2.50)
*Acinetobacter* spp.	1 (0.83)
*Pseudomonas* spp.	7 (5.83)
*Burkholderia* spp.	2 (1.67)
*Aeromonas* spp.	1 (0.83)
**Fungus**	3 (2.50)
**Others**	9 (7.50)
**Culture-negative**	12 (10.00)

**Figure 3:**
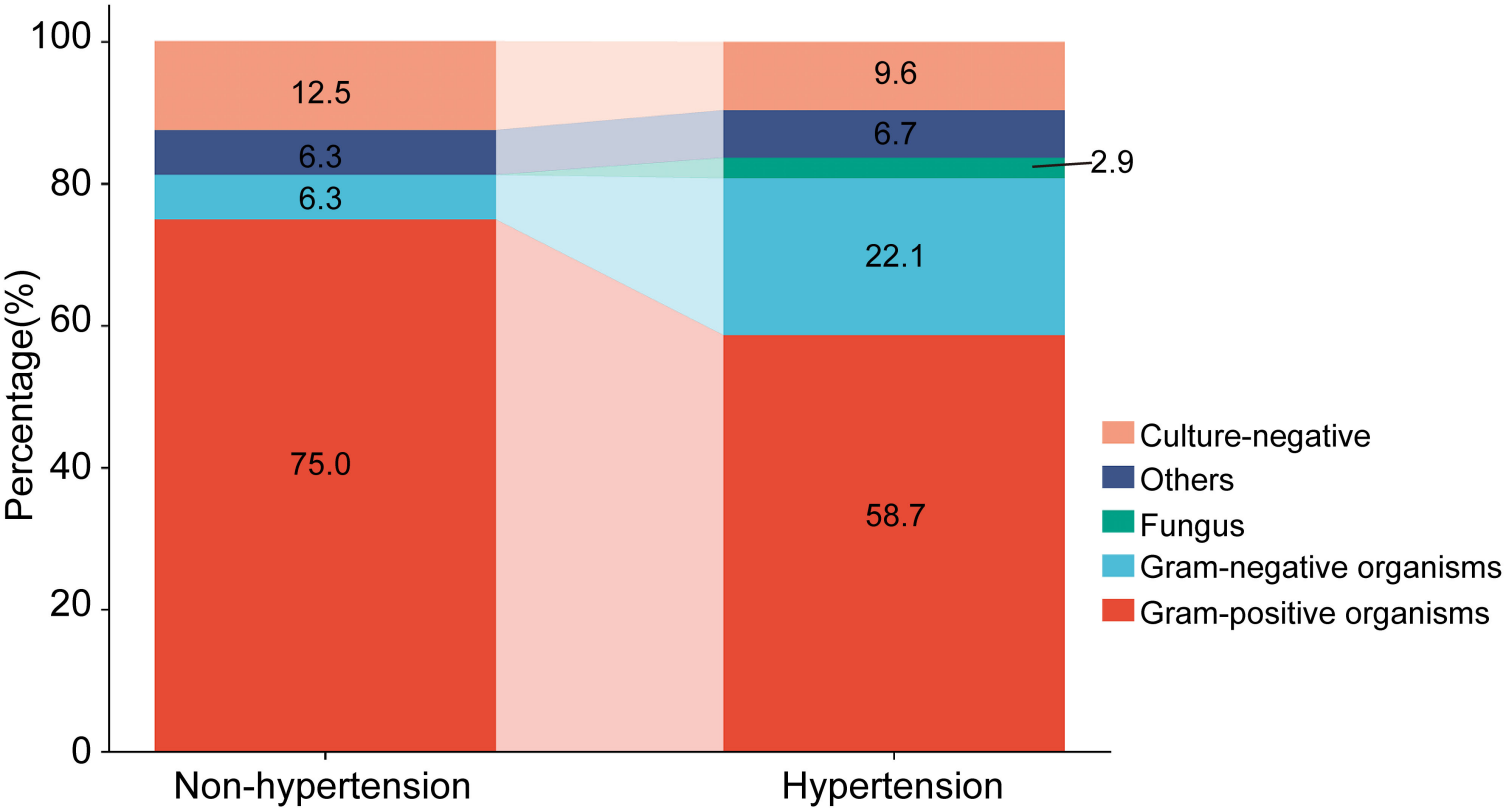
Distribution of causative microorganisms of PDAP according to hypertension status.

Further comparison of pathogen distribution between hypertensive and non-hypertensive patients (**Figure 3**) revealed that gram-positive organisms were the predominant pathogens in both groups, although their proportions differed. The proportion of gram-positive organisms was higher in the non-hypertensive group (75.0%) compared with the hypertensive group (58.7%). In contrast, gram-negative organisms were more frequently observed in hypertensive patients (22.1%) than in non-hypertensive patients (6.3%). No significant differences were observed between groups in the proportions of fungal infections, other rare pathogens, or culture-negative cases, and the overall distributions were generally similar.

## 4 Discussion

Based on a PD cohort with up to 18 years of follow-up, this study systematically evaluated the association between hypertension and the risk of PDAP. Our findings demonstrate that hypertension is highly prevalent among PD patients (55.1%) and is significantly associated with an increased incidence of PDAP. Although the two groups were generally comparable in terms of age, dialysis modality, and peritoneal transport characteristics, hypertensive patients experienced substantially more peritonitis episodes during follow-up and were more likely to transition to hemodialysis. In addition, baseline nutritional and hematologic indicators were consistently lower in the hypertensive group, suggesting reduced physiological reserve and increased susceptibility to infection. Importantly, competing risk analyses confirmed that hypertension remained an independent risk factor for PDAP even after adjustment for major confounders. These findings highlight the critical role of blood pressure status in PD populations and suggest that hypertension may be a key clinical determinant of peritoneal membrane homeostasis and immune vulnerability.

In this study, hypertension emerged as the strongest independent risk factor for PDAP, conferring an approximately fourfold increase in risk across both multivariable logistic regression and Fine–Gray competing risk models. Several hypotheses may explain the observed association, involving hypertension-related peritoneal microvascular injury, immune dysregulation, chronic systemic inflammation, and impairment of gut barrier function. Hypertension is known to induce widespread microvascular disease and endothelial dysfunction, and chronic exposure of peritoneal capillaries to elevated intravascular pressure may lead to hypoperfusion, increased endothelial permeability, and local tissue hypoxia (Wei et al., 2021). These changes may compromise peritoneal barrier integrity and facilitate bacterial translocation into the peritoneal cavity. Concurrently, increased oxidative stress and hypoxic conditions may impair the bactericidal capacity of neutrophils and macrophages. This mechanistic pathway aligns with observations by Ghamari et al., who reported that peritoneal or mesenteric hypoperfusion can result in tissue ischemia and functional impairment, leading to impaired innate immune responses and increased susceptibility to infection (Ghamari et al., 2024). Moreover, hypertension is frequently accompanied by chronic low-grade inflammation, characterized by elevated levels of proinflammatory cytokines such as IL-6 and TNF-α (Patrick et al., 2021). This persistent inflammatory burden may suppress the migratory and phagocytic responses of peritoneal immune cells, thereby weakening antimicrobial defense. In addition, hypertension has been closely linked to gut microbiota dysbiosis, including an increased abundance of Enterobacteriaceae and reduced production of short-chain fatty acids, which can disrupt intestinal barrier integrity and increase the risk of bacterial translocation (Yang et al., 2023). This mechanism is consistent with our observation of a higher proportion of gram-negative infections among hypertensive patients.

Our study further indicates that hypertensive patients exhibit a constellation of unfavorable baseline physiological characteristics at the initiation of PD that may collectively contribute to increased PDAP susceptibility. Notably, nutritional status was poorer in the hypertensive group, as evidenced by significantly lower serum albumin and transferrin saturation levels, alongside reduced hemoglobin and hematocrit. Hypoalbuminemia not only reflects protein malnutrition but also serves as a surrogate marker of systemic inflammation and altered capillary permeability, and has been well established as a strong predictor of peritonitis risk (Zha et al., 2025). Meanwhile, iron metabolism disorders and anemia may impair energy metabolism and oxidative killing capacity of immune cells, thereby reducing bacterial clearance (Noppakun et al., 2020). In addition, lower serum calcium and phosphorus levels observed in hypertensive patients remained statistically significant protective factors in multivariable analyses. These findings may reflect poorer nutritional and metabolic status associated with increased infection risk. An N et al. reported that PD patients often exhibit a chronic microinflammatory state characterized by elevated IL-6, TNF-α, and C-reactive protein levels, which may further disrupt calcium–phosphorus homeostasis and compromise barrier integrity by affecting epithelial cell adhesion, immune cell signaling, and cytoskeletal dynamics (An et al., 2023). Alterations in mineral metabolism may also modulate immune cell activation thresholds and phagocytic efficiency, leading to delayed or insufficient responses during the early stages of infection (Anghel et al., 2023). Furthermore, the lower alanine aminotransferase (ALT) levels observed in the hypertensive group may reflect impaired hepatic metabolic capacity or reduced overall metabolic reserve. As demonstrated by He Y et al., the liver plays a central role in protein and energy metabolism as well as in maintaining antioxidant and immune homeostasis. Collectively, these baseline differences in nutritional, metabolic, and inflammatory profiles suggest that hypertensive patients enter PD in a state of compromised immune defense, physiological reserve, and peritoneal homeostasis, which together form the biological basis for their increased PDAP risk.

Interestingly, despite the well-established association between hypertension and metabolic abnormalities in the general population, we observed no significant differences in triglyceride, total cholesterol, or LDL-cholesterol levels between hypertensive and non-hypertensive patients. This finding may reflect the unique pathophysiological characteristics of hypertension in PD populations, where volume overload, chronic inflammation, uremia-related factors, and nutritional status may play a more prominent role than classical metabolic syndrome (Stepanova, 2024). In addition, lipid metabolism in PD patients is influenced by multiple dialysis-related and clinical factors, which may attenuate differences attributable to hypertension alone (Lluesa et al., 2022). Consistent with this interpretation, nutritional and mineral metabolism parameters showed stronger associations with PDAP risk than conventional lipid markers in our cohort.

The present study also highlights the adverse impact of hypertension on long-term PD maintenance. During a median follow-up of approximately 46 months, hypertensive patients exhibited significantly shorter follow-up durations, which in PD research is often considered an indirect indicator of reduced dialysis vintage or impaired technique survival (Flythe and Watnick, 2024). Although peritonitis treatment outcomes, including cure rate, catheter removal, and mortality, did not differ significantly between groups, hypertensive patients experienced a higher overall incidence of peritonitis and a significantly increased likelihood of transfer to hemodialysis. Patrick G. Lan et al. demonstrated that cumulative infectious injury is a major driver of PD technique failure, which remains one of the leading causes of forced transition to hemodialysis (Lan et al., 2016). Given their higher susceptibility to peritonitis, hypertensive patients may be exposed earlier to peritoneal structural damage and ultrafiltration failure, ultimately resulting in higher rates of technique failure. Moreover, differences in post–technique failure trajectories further underscore the clinical importance of preventing infection-related technique failure. Sheru K. Kansal et al. reported that patients who transitioned directly to in-center hemodialysis after technique failure had a significantly higher risk of mortality compared with those who transitioned to home hemodialysis, suggesting that factors promoting forced conversion to hemodialysis—such as recurrent peritonitis—may amplify adverse outcomes (Kansal et al., 2019). Technique failure in PD is generally regarded as the cumulative result of multiple factors, including recurrent peritonitis, progressive peritoneal membrane damage, and declining physiological reserve (Kim et al., 2025). Taken together, hypertension appears to be not only a key risk factor for PDAP but also a critical determinant of long-term PD technique survival, operating through increased infection burden, accelerated peritoneal membrane exhaustion, and heightened risk of technique failure.

We further compared the microbiological profiles of peritonitis episodes according to blood pressure status to determine whether hypertension was associated with pathogen-specific differences. Overall, gram-positive organisms remained the predominant causative pathogens (60.8%), with Streptococcus species accounting for 39.2% of all cases, consistent with previous reports indicating the continued dominance of gram-positive bacteria in the PDAP pathogen spectrum. A five-year retrospective analysis from Anhui Province by Min Zhang et al. similarly identified Staphylococcus and Streptococcus species as the leading pathogens in PDAP (Zhang et al., 2025). Gram-negative organisms accounted for 19.2% of cases in our cohort, which is comparable to the 20–30% range reported in prior studies, while fungal peritonitis remained relatively uncommon (2.5%) but clinically significant due to its poor prognosis (Song et al., 2022). Notably, stratification by blood pressure status suggested potential differences in pathogen distribution. In non-hypertensive patients, peritonitis was predominantly caused by gram-positive organisms (75.0%), a pattern consistent with classic PDAP epidemiology and likely related to touch contamination by skin-colonizing bacteria during exchange procedures (Kim et al., 2025). In contrast, hypertensive patients appeared to show a more heterogeneous microbiological profile, characterized by a reduced proportion of gram-positive organisms (58.7%) and a higher prevalence of gram-negative infections. This finding may reflect hypertension-associated alterations in host defense and systemic inflammation. Previous studies have shown that hypertension is associated with gut microbiota dysbiosis and impaired intestinal barrier function, which may facilitate translocation of gram-negative bacteria and increase susceptibility to infection (Guo et al., 2024; Nguyen et al., 2024). Together, these observations suggest that these findings raise the possibility that hypertension may be associated with differences in microbiological spectrum; however, this observation should be interpreted cautiously because of the limited number of episodes.

Taken together, our findings underscore the clinical importance of intensified blood pressure management in PD populations. Hypertension not only significantly increases PDAP risk but also exerts an independent effect after adjustment for nutritional, metabolic, and inflammatory confounders, suggesting that blood pressure abnormalities per se may directly impair peritoneal membrane integrity and local immune defense (Zhao et al., 2024). Furthermore, hypertensive patients are characterized by lower albumin and hemoglobin levels and disturbed mineral metabolism, indicating diminished physiological reserve and heightened infection susceptibility (Xu et al., 2025). Therefore, blood pressure control in PD patients should not be viewed solely as a strategy for cardiovascular risk reduction but should also be integrated into comprehensive infection prevention frameworks. In clinical practice, optimization of antihypertensive regimens, meticulous volume management, and targeted nutritional and mineral metabolism support may synergistically reduce PDAP risk and improve patient outcomes.

Compared with previous studies, the strengths of this work include the extended follow-up period and the application of competing-risk analyses to evaluate the association between hypertension and PDAP. Nevertheless, several limitations should be acknowledged. First, this was a retrospective single-center study, which may limit the generalizability of the findings. Second, hypertension was defined using baseline clinical information, and longitudinal data regarding blood pressure control, ambulatory blood pressure monitoring, blood pressure variability, resistant hypertension, volume status, and changes in antihypertensive therapy were unavailable. Because blood pressure in PD patients is dynamic and often volume-dependent, some degree of exposure misclassification during follow-up cannot be excluded. Third, although multivariable adjustment and competing-risk analyses were performed, the hypertension and non-hypertension groups differed in several baseline characteristics, including diabetes prevalence, nutritional indicators, and mineral metabolism parameters. In addition, propensity score–based approaches were not performed in the present study. Fourth, detailed longitudinal medication data, including antihypertensive agents, lipid-lowering therapies, immunosuppressive drugs, corticosteroids, phosphate binders, vitamin D preparations, and erythropoiesis-stimulating agents, were not consistently available and could not be incorporated into the analyses. Future multicenter prospective studies with dynamic blood pressure assessment and more comprehensive clinical data are needed to validate these findings and further clarify the mechanisms underlying the observed association.

## 5 Conclusion

In this retrospective cohort study, baseline hypertension was independently associated with a higher risk of PDAP, even after adjustment for multiple clinical and laboratory factors and consideration of competing events. Hypertensive patients also exhibited less favorable long-term PD outcomes, including a higher likelihood of transfer to hemodialysis. These findings suggest that blood pressure status may represent an important marker of PDAP susceptibility and should be considered in risk stratification and long-term management of PD patients.

## Data Availability

The original contributions presented in the study are included in the article/supplementary material. Further inquiries can be directed to the corresponding author.
